# Impact of weight variation on the microbiome of yak dams and calves

**DOI:** 10.3389/fmicb.2024.1465992

**Published:** 2024-09-18

**Authors:** Hongzhuang Wang, Wangdui Basang, Zhandui Pingcuo, Nan Jiang, Guangming Sun, Shah Nawaz, Yangji Cidan, Yang Liu, Yanbin Zhu, Dunzhu Luosang

**Affiliations:** ^1^State Key Laboratory of Hulless Barley and Yak Germplasm Resources and Genetic Improvement, Lhasa, China; ^2^Institute of Animal Husbandry and Veterinary Medicine, Tibet Academy of Agriculture and Animal Husbandry Science, Lhasa, China; ^3^Department of Anatomy, Faculty of Veterinary Science, University of Agriculture, Faisalabad, Pakistan

**Keywords:** yak, calf, weight, microbiota, oxidative resistance

## Abstract

**Introduction:**

Limited information exists regarding the microbiome composition of yak calves of varying weights. Therefore, this study aimed to investigate the microbiomes of mother-calf pairs with different weight profiles.

**Methods:**

Fecal and blood samples were collected from both lower-weight (CB) and higher-weight (HB) yak calves, along with their corresponding female yaks (CA, HA).

**Results:**

The results revealed significantly higher levels of T-AOC (total antioxidant capacity) and GSH-Px (glutathione peroxidase) in HB animals (*p* < 0.001). Sequencing yielded 652,181 and 643,369 filtered reads in female and calf yaks, respectively. Alpha diversity analysis indicated that Chao1, Faith_pd, and Observed species were significantly higher in CA compared to HA (*p* < 0.01). Furthermore, nine genera were notably different between HA and CA yaks, including Avispirillum, Fimenecus, CAG-1031, Odoribacter 865974, and Jeotgalicoccus A 310962. Compared to CB yaks, CA animals exhibited significant differences in one phylum and six genera, including CAG-485 (*p* < 0.05), CAG-83 (*p* < 0.01), *Copromorpha* (*p* < 0.01), *Phocaeicola* A 858004 (*p* < 0.05), and UBA2253 (*p* < 0.05).

**Conclusion:**

In summary, higher-weight yak calves demonstrated increased oxidative resistance, and weight profiles were linked to the microbiomes of both female yaks and their calves. These findings offer valuable insights for optimizing yak breeding practices in high-altitude regions.

## Introduction

The Qinghai-Tibet Plateau, often referred to as the “Third Pole” of the world, spans the geographic coordinates of approximately 26°00′ to 39°47′ north latitude and 73°19′ to 104°47′ east longitude. This region is characterized by its oxygen-deficient atmosphere, extreme cold temperatures, and intense ultraviolet radiation exposure (Liu et al., 2022). The yak is a ruminant bovine species primarily inhabiting in plateau regions. This ancient breed has inhabited the Tibetan Plateau for thousands of years and has developed a strong adaptability to the region’s harsh ecological conditions (Wan et al., 2021; Ding et al., 2023), which provide nourishing meat and milk products, serve as transportation, and produce valuable resources such as fueling dung, fur, and medicinal materials, with their horns being utilized by plateau herdsmen (Li et al., 2020). Over 16 million of these economically significant animals reside in China’s western regions, comprising 90% of the global population of this species (Dong et al., 2023; Shang et al., 2022). Yak breeding serves as a primary industry in the remote plateau regions, making efficient farming practices crucial and significant for local communities.

The gut microbial communities consist of billions of microorganisms, including viruses, bacteria, fungi, and protozoa (McCallum and Tropini, 2024; Xi et al., 2021), which are also considered microbial organs making contributions to the digestion and metabolism, gut barrier development and immune functions. The microbiome, a complex microbial organ in the host’s gastrointestinal tract, significantly influences the host’s metabolic system (Islam et al., 2023; Xie et al., 2024). Previous studies have reported that microbiome was highly associated with child growth, and become a devasting global health issue with long-lasting consequences such as stunted growth and abnormal development and animal growth (Blanton et al., 2016; Wang et al., 2023). In many animals, the microbiome is crucial for survival, as it supports essential functions such as digestion, immunity, and behavior through mutualistic relationships (Perlman et al., 2022; Ren et al., 2023). The mother’s microbiota significantly influences the establishment of an infant’s microbiome in early life during delivery, maternal environmental exposures, and subsequent environmental factors, all of which play a crucial role in shaping the infant’s future health (Bogaert et al., 2023; Browne et al., 2022). Furthermore, previous experiments have also confirmed that microbiota enhances the growth performance of chicks and young mice (Pronovost et al., 2023; Zhang et al., 2022). However, there is limited information available regarding the microbiome of yak calves with varying weights. Therefore, we conducted this research to investigate the mother-calf microbiome of yaks with different weights.

This study is crucial as it sheds light on the previously unexplored relationship between weight profiles and microbiomes in yaks, especially between dams and calves. This study also provides valuable insights into the intricate relationship between weight profiles and microbiomes in yak dams and calves. The study also aims to correlate the levels of T-AOC and GSH-Px with the weight and microbiome profiles of the calves, to elucidate the potential mechanisms through which weight variation influences oxidative resistance and overall health in livestock. By examining this connection, the research contributes to a deeper understanding of how maternal and offspring microbiomes influence growth and health outcomes in livestock. The findings of this study could have significant implications for improving breeding strategies, optimizing livestock management, and potentially informing interventions to enhance the health and productivity of yaks and other similar species in the future.

## Materials and methods

### Sample collection

**Figure 1:**
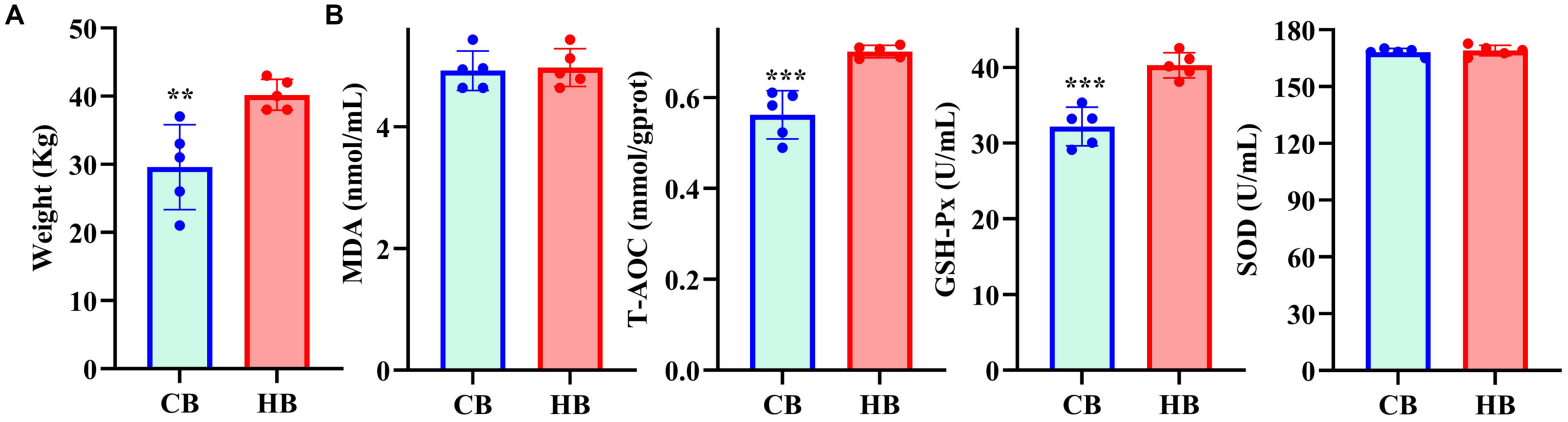
The weight of yak calves **(A)** and oxidation resistance **(B)** in different groups.

Fecal and blood samples were obtained from 1 month-old yak (Figure 1) calves with lower (CB) and higher (HB) weights, along with their corresponding female yaks (CA, HA), from a local breeding company in Lhasa, China, in December 2023.

### Oxidation resistance analysis

Each blood sample underwent centrifugation at 3,800 g for 15 min to isolate serum, which was then stored at −20°C for subsequent analysis. The antioxidant capacity (T-AOC) of yak calves was determined using a commercial Assay kit using a colorimetric method to assess antioxidant levels in biological samples. Samples were diluted and mixed with standard antioxidants provided in the kit. After incubation and the addition of a chromogenic reagent, absorbance was measured at 405 nm. Results were calculated using a standard curve and expressed as mM of Trolox equivalents per sample volume. Oxidative resistance was assessed by measuring the malondialdehyde (MDA), superoxide dismutase (SOD), and glutathione peroxidase (GSH-Px) levels. Malondialdehyde levels were determined spectrophotometrically, superoxide dismutase activity was assessed using a commercially available assay kit, and glutathione peroxidase levels were measured via colorimetric methods. All kits were procured from Jiancheng Bioengineering Research Institute Co., Ltd. (China).

### Microbiota sequencing

The total DNA of mother yaks (HA, HB) and calf yaks (CA, CB) was extracted using a soil DNA kit (Omega Bio-Tek, United States). Subsequently, the quality of the yak DNA was assessed using a NanoDrop eight spectrophotometer (Thermo Scientific, United States) and 0.8% agarose gel electrophoresis (He et al., 2023). The 16S rRNA region V3–V4 of mother and calf yaks were amplified by employing a universal primer pair (338F: 5’-ACTCCTACGGGAGGCAGCA-3; 806R: 5’-GGACTACHVGGGTWTCTAAT-3′) (Zeng and An, 2021). The amplified products were purified via GeneJET (Thermo Scientific, United States) for constructing sequencing library through Hieff NGS^®^ OnePot kit (Yeasen, China). At last, sequencing of yak microbiomes was performed on Illlumina MiSeq platform at Bioyi Biotechnology Co., Ltd. (Wuhan, China).

### Bioinformatic analysis of yaks

The raw data from mother and calf yaks was filtered to create the feature form of amplicon sequence variants by QIIME2 (Mohsen et al., 2022). The ASVs were aligned using MAFFT to generate a systematic form (Nakamura et al., 2018), and Venn diagram was constructed using the R package to illustrate the shared ASVs between HA and CA, as well as HB and CB, respectively (Xiao et al., 2022). Richness and evenness comparisons of ASVs between HA and CA, as well as HB and CB yaks, were conducted by plotting ranked abundance curves. Alpha diversity metrics, including Chao1, Faith_pd, Shannon, Goods_coverage, Observed_species, Pielou_e, and Simpson, were analyzed using QIIME2. To explore the configurational variation between HA and CA, and HB and CB ruminants, we performed beta diversity analysis of principal coordinate analysis (Shi et al., 2020) and nonmetric multidimensional scaling (Feng et al., 2023). The differential abundance of taxa between the HA and CA groups, as well as the HB and CB groups, was assessed using Linear Discriminant Analysis Effect Size (LEfSe) and *t*-tests (Wang et al., 2024). Microbial functions of HA and CA, as well as HB and CB yaks, were analyzed using PICRUSt2 with reference to the MetaCyc and KEGG databases.

## Statistical analysis

Results of HA, CA, HB and CB ruminants were examined through student’s *t*-test utilizing SPSS (IBM, 26.0), and presented as average value ± SD, and significance is considered when *p* < 0.05.

## Results

### Body weights and antioxidant levels of yaks

The average weight of CB yaks (29.6 kg) was significantly lower than that of HB yaks (40.2 kg) (Figure 1A). Serum analysis revealed significantly higher levels of T-AOC (*p* < 0.001) and GSH-Px (*p* < 0.001) in HB animals (Figure 1B).

### Microbiome analysis of mother and calf animals

**Figure 2:**
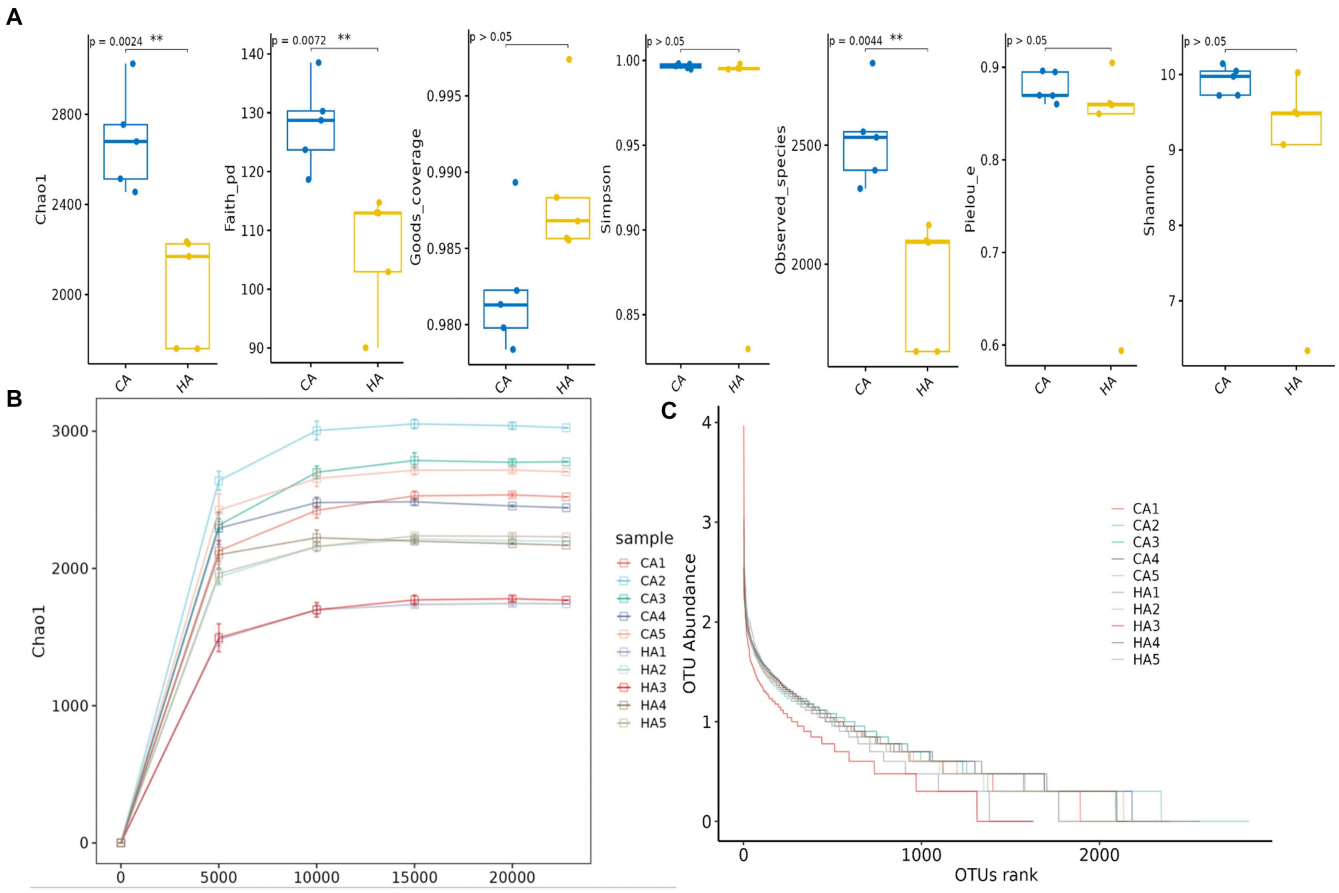
Alpha diversity analysis of female yaks in different groups. **(A)** Indexes, **(B)** rarefaction curve, **(C)** rank abundance curve.

**Figure 3:**
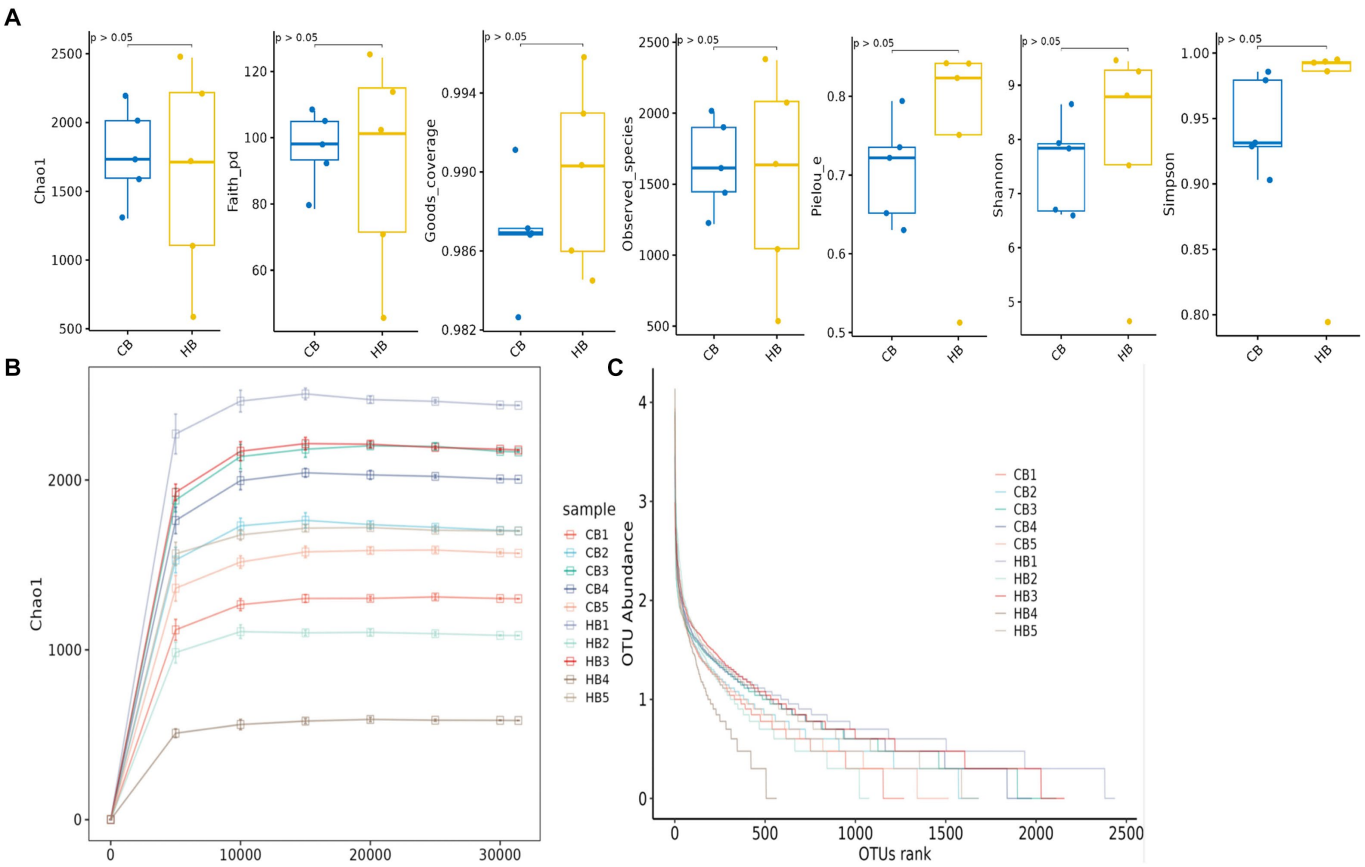
Alpha diversity analysis of yak calves in different groups. **(A)** Indexes, **(B)** rarefaction curve, **(C)** rank abundance curve.

**Figure 4:**
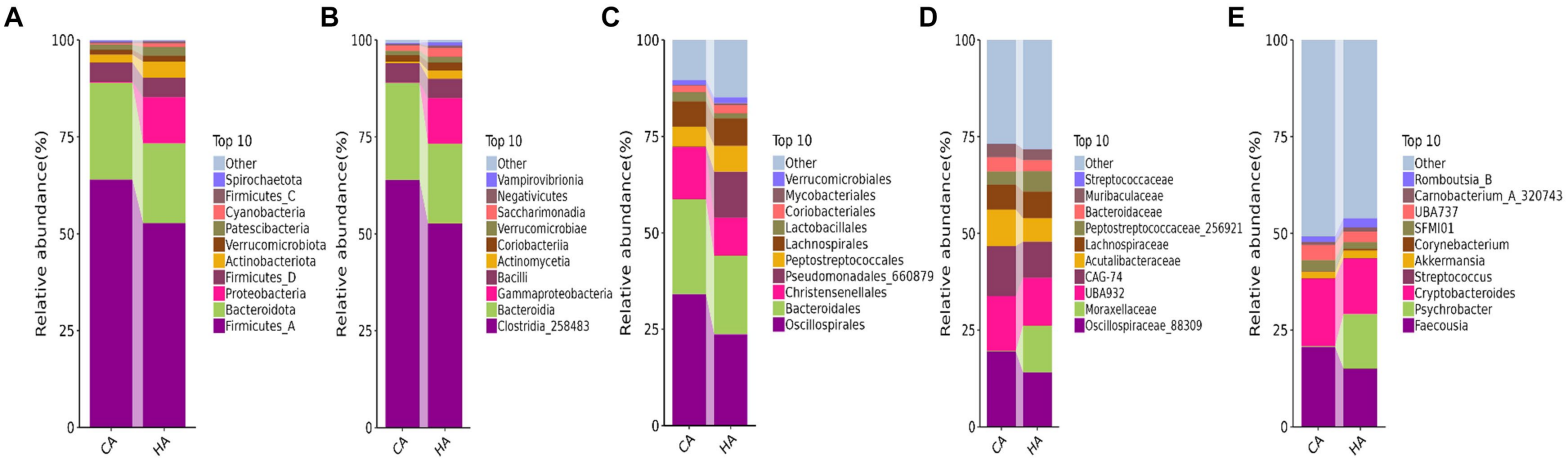
Analyzing the intestinal flora structure female yaks in different taxa. **(A)** Phylum, **(B)** class, **(C)** order, **(D)** family, **(E)** genus.

In female and calf yaks (Table 1), there were over 67,000 (CA), 49,000 (HA), 63,000 (CB), and 64,000 (HB) raw sequences, and 62,000 (CA), 46,000 (HA), 60,000 (CB), and 59,000 (HB) filtered sequences. Alpha diversity analysis revealed that Chao1, Faith_pd, and Observed_species were significantly higher (*p* < 0.01) in CA yaks compared to HA yaks (Table 2; Figure 2A). However, no significant differences were observed between HB and CB yaks in terms of these indices (Figure 3A). The rarefaction curves in all ruminant groups appeared as nearly horizontal lines (Figures 2B, 3B), indicating that the current sequencing depth was adequate. Furthermore, the rank abundance curves in HA, CA, HB, and CB groups all displayed smooth horizontal curves (Figures 2C, 3C), suggesting a high uniformity in the current flora composition of yaks. At the phylum level of female yaks, Firmicutes_A (52.77%), Bacteroidota (20.57%), and Proteobacteria (11.86%) were the dominating phyla in HA, while Firmicutes_A (63.98%), Bacteroidota (24.98%) and Firmicutes_D (4.95%) were the main phyla in CA (Figure 4A). At the class level, Clostridia_258483 (52.74%), Bacteroidia (20.58%), and Gammaproteobacteria (11.67%) were the staple classes in HA, while Clostridia_258483 (63.94%), Bacteroidia (24.98%) and Bacilli (4.95%) were the staple classes in CA (Figure 4B). At the order level, Oscillospirales (23.73%), Bacteroidales (20.37%) and Pseudomonadales_660879 (11.91%) were the prime orders in HA, while Oscillospirales (34.14%), Bacteroidales (24.58%) and Christensenellales (13.49%) were the prime orders in CA (Figure 4C). At the family level, Oscillospiraceae_88309 (14.11%), UBA932 (12.33%) and Moraxellaceae (12.06%) were mainly examined in HA, while Oscillospiraceae_88309 (19.43%), UBA932 (14.24%) and CAG-74 (12.87%) were mainly examined in CA (Figure 4D). At the genus level, *Faecousia* (15.02%), *Cryptobacteroides* (15.31%) and *Psychrobacter* (14.16%) were the stable genera in HA, while *Faecousia* (20.64%), *Cryptobacteroides* (17.41%) and UBA737 (3.93%) were the stable genera in CA (Figure 4E).

**Table 1:** Sequencing data information of female and calf yaks.

Samples	Input	Filtered	Denoised	Merged	Non-chimeric	Non-singleton
CA1	79,191	74,019	69,211	49,048	43,474	43,112
CA2	78,342	73,117	67,263	42,559	37,213	36,571
CA3	77,375	72,554	68,010	46,729	41,027	40,529
CA4	67,168	62,783	57,423	34,517	31,164	30,745
CA5	73,081	68,540	63,291	42,226	37,376	36,955
HA1	73,390	68,744	65,527	51,771	45,865	45,642
HA2	67,440	63,256	59,641	45,294	40,958	40,665
HA3	63,624	59,780	56,537	44,419	41,723	41,505
HA4	49,183	46,250	42,591	26,803	24,347	23,951
HA5	67,612	63,138	59,556	44,433	40,524	40,264
CB1	64,478	60,587	58,163	48,578	41,537	41,389
CB2	69,069	64,687	61,181	48,204	38,475	38,147
CB3	74,725	69,783	65,619	47,901	43,423	43,077
CB4	70,676	66,219	62,352	47,043	39,150	38,892
CB5	70,429	66,007	63,014	52,751	47,207	47,010
HB1	63,410	59,454	55,180	38,444	33,517	33,055
HB2	67,620	63,433	60,875	50,585	38,389	38,272
HB3	70,720	66,277	62,365	45,454	37,591	37,169
HB4	64,507	60,486	59,148	54,667	50,313	50,270
HB5	71,175	66,436	62,587	45,698	38,943	38,706

**Table 2:** Alpha diversity analysis of yaks in different groups.

Sample	Chao1	Faith pd	Goods coverage	Observed species	Pieloue	Shannon	Simpson
CA1	2513.467837	123.6939283	0.981296418	2317.9	0.869596076	9.720861177	0.996722034
CA2	3024.733427	138.5240344	0.978378378	2844.9	0.869415436	9.975806249	0.995637341
CA3	2754.36845	118.6390105	0.979789057	2556.5	0.896053241	10.14327263	0.998225803
CA4	2455.34182	128.7125304	0.989321028	2394.7	0.894845704	10.0452013	0.99793998
CA5	2679.41716	130.2878843	0.982263239	2533.4	0.86023156	9.726513593	0.994828114
HA1	1760.380056	90.04198729	0.988319051	1632.8	0.849684276	9.068772085	0.994756542
HA2	2225.065825	112.954356	0.985559218	2092	0.859403911	9.479785943	0.995322081
HA3	1759.539369	102.9579706	0.986816084	1,633	0.594045751	6.340421835	0.829832148
HA4	2169.342575	114.7329726	0.997402769	2164.8	0.904885395	10.02614645	0.998055731
HA5	2234.462573	113.0062686	0.985642716	2099.8	0.86091205	9.501040934	0.995386831
CB1	1302.178786	78.50230447	0.991078884	1219.3	0.651598033	6.680058661	0.928670701
CB2	1732.972975	98.09163012	0.987150077	1614.1	0.735351223	7.836270192	0.979335015
CB3	2191.645068	108.0825097	0.982627994	2013.5	0.721799593	7.92207332	0.931429622
CB4	2012.564727	104.8840538	0.986816084	1900.1	0.794066697	8.648853147	0.985721251
CB5	1595.162537	93.28245017	0.986930345	1446.7	0.630194649	6.616104676	0.903153886
HB1	2472.009209	124.170931	0.985985498	2,374	0.841998965	9.441419798	0.992554036
HB2	1107.7071	71.48705164	0.992981762	1046.4	0.751026776	7.533684364	0.986175709
HB3	2218.354729	114.9875933	0.984544056	2082.9	0.841782629	9.280123022	0.995212338
HB4	589.8400392	46.03565591	0.995833883	538.6	0.512563847	4.650498131	0.794495228
HB5	1711.620424	101.2296307	0.990309822	1635.1	0.823268207	8.788508186	0.993385299

**Figure 5:**
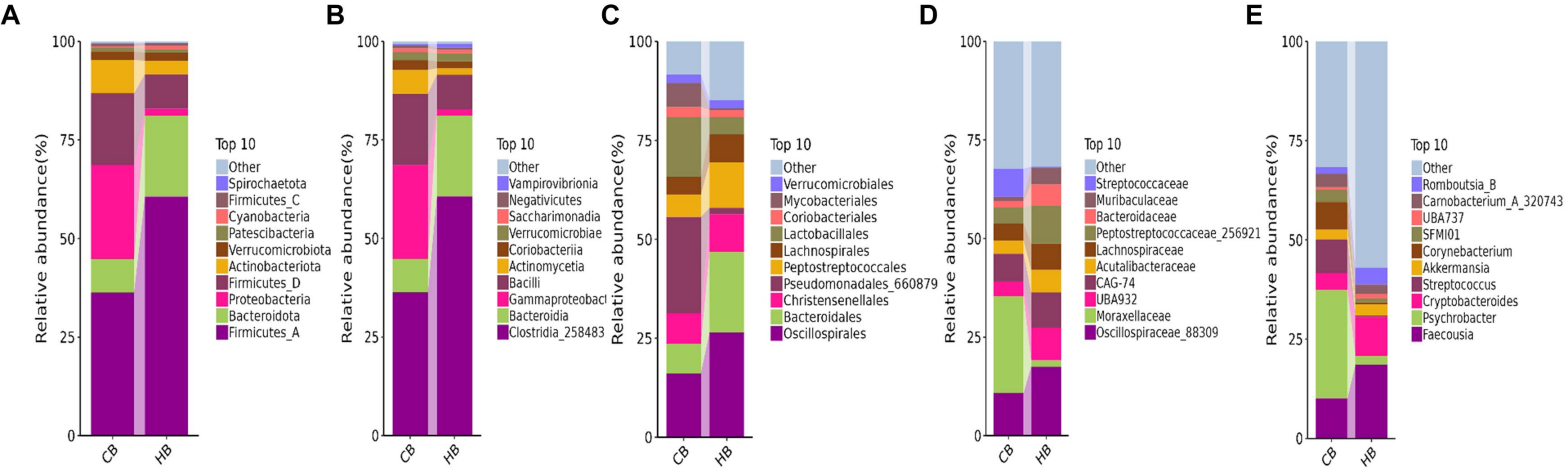
Analyzing the intestinal flora structure yak calves in different taxa. **(A)** Phylum, **(B)** class, **(C)** order, **(D)** family, **(E)** genus.

At the phylum level of yak calves, Firmicutes_A (60.64%), Bacteroidota (20.56%), and Firmicutes_D (8.74%) were the primary phyla in HB, while Firmicutes_A (36.32%), Proteobacteria (23.88%) and Firmicutes_D (18.11%) were the primary phyla in CB (Figure 5A). At the class level, Clostridia_258483 (60.64%), Bacteroidia (20.56%), and Bacilli (8.74%) were the dominating classes in HB, while Clostridia_258483 (36.31%), Gammaproteobacteria (23.82%) and Bacilli (18.11%) were the dominating classes in CB (Figure 5B). At the order level, Oscillospirales (26.51%), Bacteroidales (20.31%), and Peptostreptococcales (11.52%) were mainly detected in HB, while Pseudomonadales_660879 (24.31%), Oscillospirales (16.17%) and Lactobacillales (15.11%) were mainly detected in CB (Figure 5C). At the family level, Oscillospiraceae_88309 (17.52%), CAG-74 (8.94%) and UBA932 (8.26%) were the prime families in HB, while Moraxellaceae (24.59%), Oscillospiraceae_88309 (10.82%) and CAG-74 (7.05%) were the prime families in CB (Figure 5D). At the genus level, *Faecousia* (18.61%), *Cryptobacteroides* (9.85%), and *Romboutsia*_B (4.22%) were the staple genera in HB, while *Psychrobacter* (27.30%), *Faecousia* (10.15%) and *Streptococcus* (8.45%) were the staple genera in CB (Figure 5E).

### The abundant species in the microbiota of yaks in dams and calves

**Figure 6:**
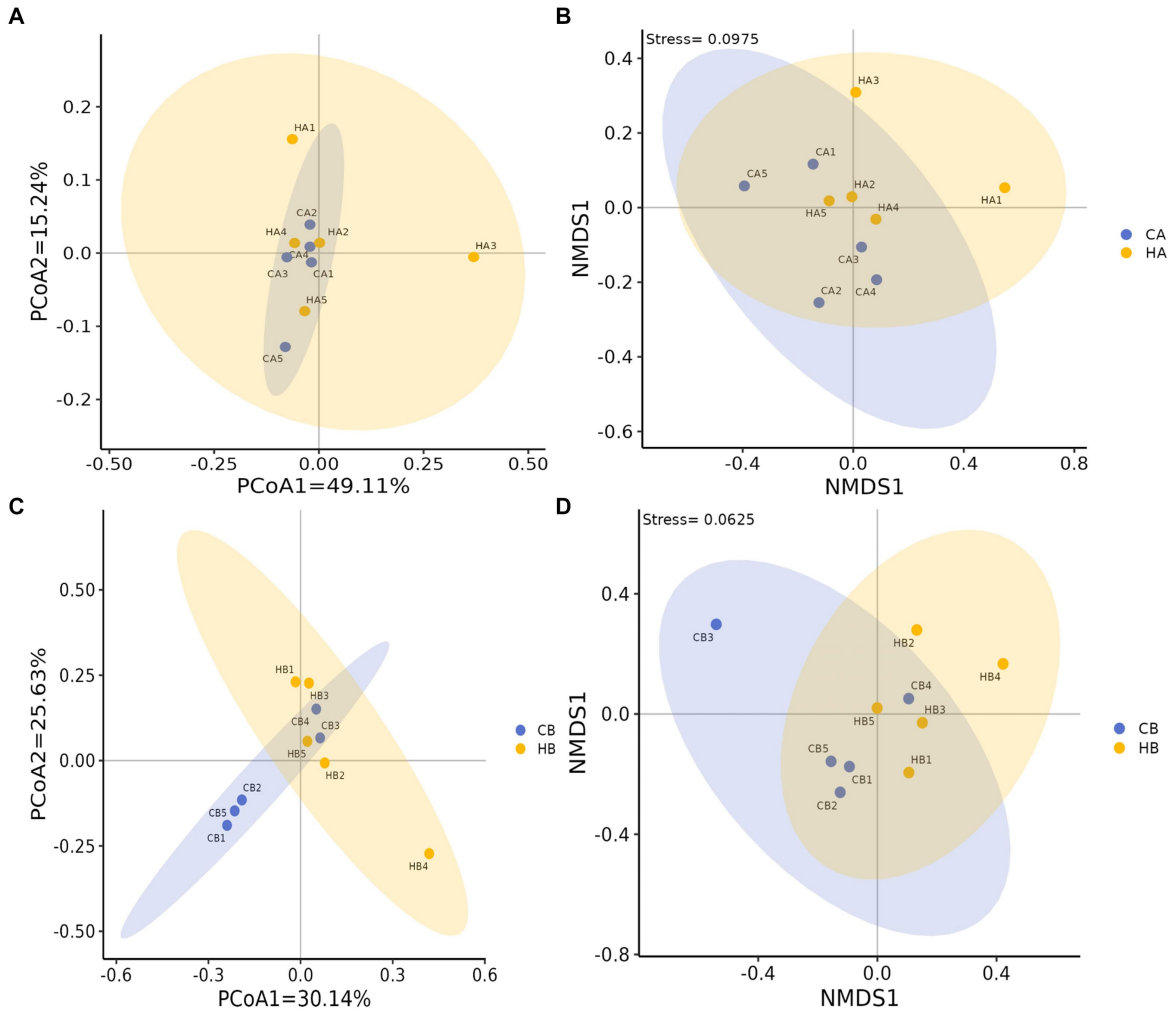
Beta diversity analysis of female yaks (**(A)** PCoA, **(B)** NMDS) and yak calves (**(C)** PCoA, **(D)** NMDS).

**Figure 7:**
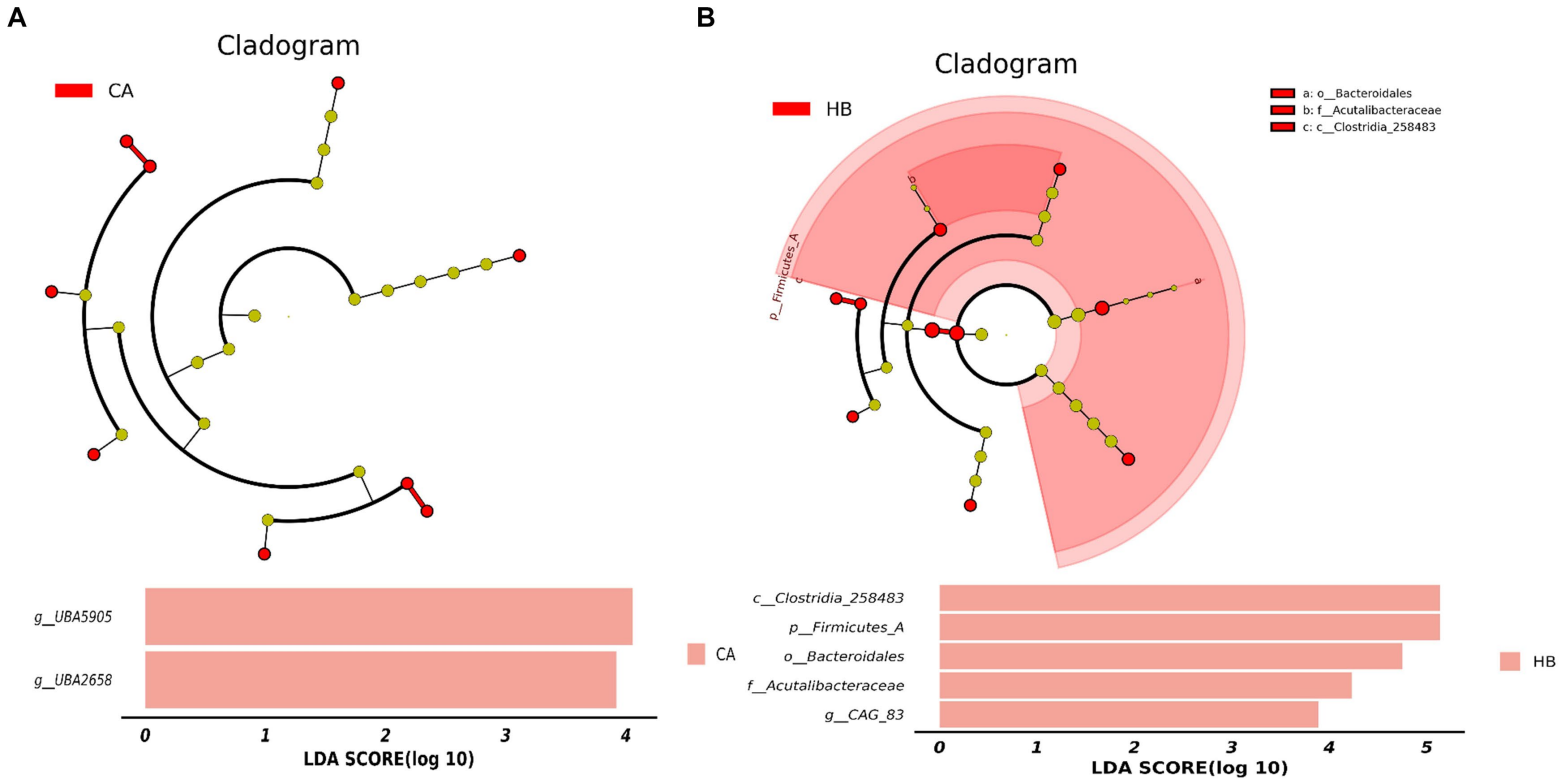
Revealing the markedly different species between different groups via LEfSe. **(A)** Phylum (HA and CA), **(B)** genus (HA and CA), **(C)** phylum (HB and CB), **(D)** genus (HB and CB).

**Figure 8:**
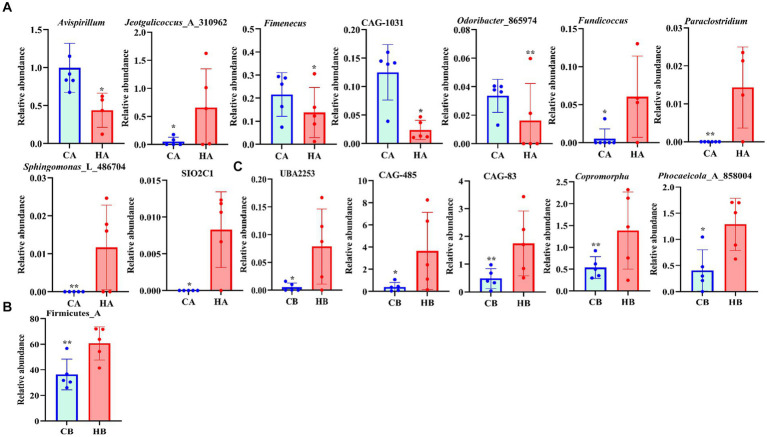
Revealing the markedly different species between different yaks via *t*-test. **(A)** Genus (CA and HA), **(B)** phylum (CB and HB), **(C)** genus (CB and HB).

Analysis of beta diversity using PCoA and NMDS revealed that the variation between CA and HA animals, as well as CB and HB yaks, was minimal. This similarity in distance suggested a close resemblance in the microbiome composition between female yaks in CA, HA, and between yak calves in CB and HB (Figure 6). LEfSe analysis revealed significant differences in genera abundance. In CA, the genera g__UBA5905 (LDA = 4.05, *p* < 0.05) and g__UBA2658 (LDA = 3.92, *p* < 0.05) were notably elevated (Figure 7A). Conversely, in HB, Clostridia_258483 (LDA = 5.13, *p* < 0.05), Firmicutes_A (LDA = 5.13, *p* < 0.05), o__Bacteroidales (LDA = 4.75, *p* < 0.05), f__Acutalibacteraceae (LDA = 4.23, *p* < 0.05), and g__CAG_83 (LDA = 3.87, *p* < 0.05) exhibited significantly higher levels (Figure 7). At the genera level, *Avispirillum*, *Fimenecus*, CAG-1031, and *Odoribacter*_865,974 were considerably higher (*p* < 0.01) in CA yaks, while *Jeotgalicoccus*_A_310,962 (*p* < 0.05), *Fundicoccus* (*p* < 0.05), *Paraclostridium* (*p* < 0.01), *Sphingomonas*_L_486704 (*p* < 0.01) and SIO2C1 (*p* < 0.05) were markedly higher in HA ruminants (Figure 8A). Compared with CB yaks, *Firmicutes_A* was significantly higher (*p* < 0.01) in HB animals (Figure 8B). CAG-485 (*p* < 0.05), CAG-83 (*p* < 0.01), *Copromorpha* (*p* < 0.01), *Phocaeicola*_A_858,004 (*p* < 0.05) and UBA2253 (*p* < 0.05) were significantly higher in HB (Figure 8C).

### Functional analysis of yaks in different groups

**Figure 9:**
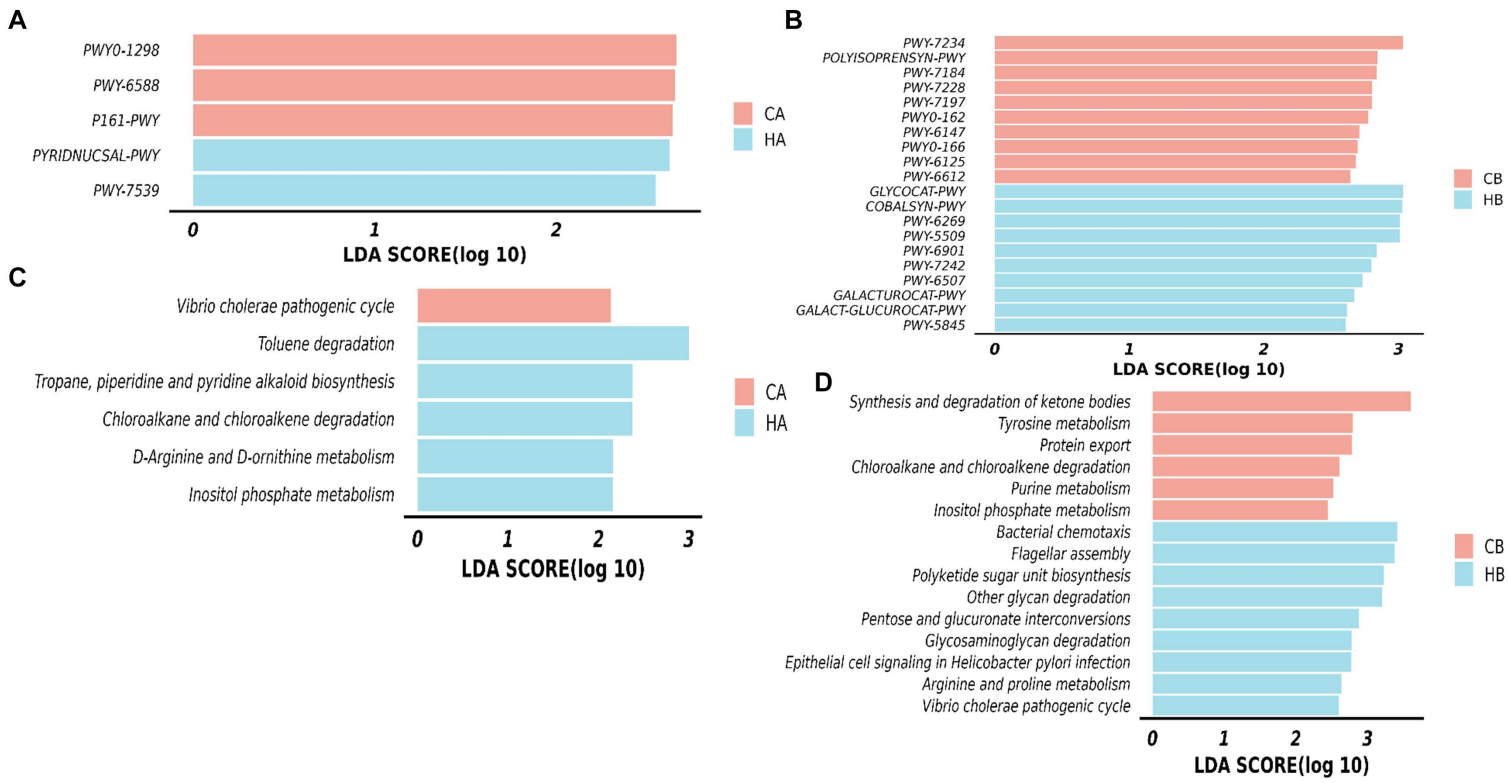
Analyzing the microbiota function between female yaks and yak calves. **(A)** MetaCyc pathways of female yaks, **(B)** KEGG pathways of female yaks, **(C)** MetaCyc pathways of yak calves, **(D)** KEGG pathways of yak calves.

The MetaCyc pathways analysis highlighted distinct patterns in female yaks. In CA yaks, pathways including P161-PWY, PWY-6588, and PWY0-1298 exhibited notably elevated levels (*p* < 0.05). Likewise, in HA animals, PWY-7539 and PYRIDNUCSAL-PWY showed significantly higher (*p* < 0.05) activity (Figure 9A). KEGG pathways of female yaks showed that pathways of chloroalkane and chloroalkene degradation (*p* < 0.05), D-Arginine and D-ornithine metabolism (*p* < 0.05), Inositol phosphate metabolism (*p* < 0.05), Toluene degradation (*p* < 0.05) and Tropane, piperidine and pyridine alkaloid biosynthesis (*p* < 0.05) were memorably higher in HA, while *Vibrio cholerae* pathogenic cycle (*p* < 0.05) was memorably higher in CA (Figure 9B). The MetaCyc pathways analysis of yak calves unveiled significant differences between HB and CB. In HB, pathways such as COBALSYN-PWY, FUC-RHAMCAT-PWY, GALACT-GLUCUROCAT-P, GALACTUROCAT-PWY, GLUCARDEG-PWY, GLYCOCAT-PWY, and numerous others (*p* < 0.05 or *p* < 0.01) were notably elevated. Conversely, in CB, pathways including FOLSYN-PWY, POLYISOPRENSYN-PWY, PWY-6125, and others (*p* < 0.05) exhibited substantially higher levels (Figure 9C). KEGG pathways of yak calves showed that pathways of arginine and proline metabolism, bacterial chemotaxis, epithelial cell signaling in *Helicobacter pylori* infection, Flagellar assembly, Glycosaminoglycan degradation, Other glycan degradation, Pentose and glucuronate interconversions, Polyketide sugar unit biosynthesis and *Vibrio cholerae* pathogenic cycle were observably higher (*p* < 0.05) in HB, while Chloroalkane and chloroalkene degradation, Inositol phosphate metabolism, Protein export, Purine metabolism, synthesis and degradation of ketone bodies and tyrosine metabolism were dramatically higher (*p* < 0.05) in CB (Figure 9D).

## Discussion

The yak holds significant economic and religious importance in the plateau regions of western China. Efficient propagation and breeding of yaks are pressing issues that require urgent attention. In this study, we specifically selected young yak calves exhibiting notable weight differences. The study revealed a clear association between weight variation in yak calves and significant differences in both microbiome composition and oxidative stress markers. Specifically, higher-weight calves were characterized by enhanced microbial richness and phylogenetic diversity, coupled with increased levels of oxidative resistance markers (T-AOC and GSH-Px). These findings suggest a multifaceted relationship where weight profiles may influence gut microbiota, which in turn could impact the calves’ oxidative stress responses and overall health. Reactive oxygen species, such as superoxide and peroxynitrite, are produced during physiological processes. Excessive ROS levels can lead to inflammatory responses and diseases by damaging biomacromolecules. However, the excessive activation of the inflammatory response inevitably harms the organism and leads to severe damage (Liu et al., 2023; Mendonça et al., 2022; Kazmi et al., 2023). T-AOC and GSH-Px are two vital antioxidant enzymes (Xie et al., 2022), the higher levels of these enzymes indicate that the HB yak calves had better oxidation resistance.

Subsequently, we conducted microbiota analysis on female yaks in HA and CA, as well as yak calves in HB and CB. Our analysis yielded 652,181 filtered reads for female yaks and 643,369 filtered reads for yak calves, respectively. Alpha diversity analysis revealed significant differences between CA and HA. The Chao1 index, a richness estimator, was employed to assess the number of observed species, providing insights into the microbial diversity within each group. The Chao1 index revealed significant differences in microbial richness between the high-weight and low-weight calves, indicating a greater number of unique species present in the microbiomes of the high-weight calves. Chao1, Faith_pd, and Observed_species were notably higher (*p* < 0.01) in CA than in HA, indicating a higher species richness in CA (Galvão et al., 2019). At different taxa, the microbiota structure of female yaks (HA, CA) and yak calves (HB, CB) were different. Firmicutes and Bacteroidota are the dominating phyla in healthy host (Jandhyala, 2015), and the ratio of Firmicutes/Bacteroidota in HA (2.34) was lower than CA (3.40), while HB (3.40) was higher than CB (2.68). The higher Firmicutes/Bacteroidota values in HB yaks were in line with obese people (Asha and Sharma, 2020; Koliada et al., 2017).

Additionally, we conducted a comparison of the yak microbiota at the phylum and genus levels. Our analysis revealed significant differences between HA and CA yaks, with 9 genera (Avispirillum, Fimenecus, CAG-1031, Odoribacter_865,974, and Jeotgalicoccus_A_310,962) showing distinctive patterns. Due to the limited available information on these genera, we can infer their potential relevance to intestinal flora homeostasis in yaks. Contrasting with CB yaks, HB animals exhibited significant differences in one phylum and six genera. Genus of CAG-485 was related to host disease resistance (Bowerman et al., 2020), and a higher abundance of *Phocaeicola* was found in healthy people (Al Bataineh et al., 2023), which may reveal that the higher weight of yak calves was related to those genera.

The microbiota difference also affected its function in yaks. Through MetaCyc and KEGG pathways analysis, we identified five and six significantly distinct pathways in female yaks, respectively. Among them, inositol phosphate is an important coordinator in nutrition metabolism (Tu-Sekine and Kim, 2022), the higher abundance of inositol phosphate HA yaks may promote the growth of its calves. Similarly, thirty-three MetaCyc pathways and fifteen KEGG pathways were observably different in yak calves, among them, Glycosaminoglycan and other glycan were considered the primary energy and nutrition for the intestinal flora (Ndeh and Gilbert, 2018), which may favor the growth of yak calves.

The positive correlation between higher weight and oxidative resistance observed in this study may be indicative of a protective mechanism facilitated by a diverse microbiome. A richer microbiota could contribute to the synthesis of metabolites that enhance antioxidant defenses, thereby reducing oxidative stress. This potential mechanism warrants further investigation, as it could reveal novel strategies for improving livestock health through microbiome modulation. To build on the current study, future research should explore the mechanistic pathways that link microbiome diversity with oxidative stress and health outcomes in yak calves. Additionally, studies that investigate these relationships under different environmental and dietary conditions would provide a more comprehensive understanding of how to optimize livestock management practices.

## Conclusion

In our study, we observed that yak calves with greater weights exhibited increased oxidation resistance, and we noted an association between yak calf weights and the microbiomes of both female yaks and yak calves. These results underscore the critical role of weight profiles in modulating the gut microbiome and oxidative stress responses, which are vital for the overall health and resilience of the calves. The findings not only contribute to our understanding of the microbiome-health axis in livestock but also offer practical implications for improving animal management practices. Future research should explore the mechanistic pathways underlying these associations and evaluate their potential impact on livestock productivity and welfare.

## Data Availability

All raw sequence data from female yaks and yak calves were deposited in the NCBI Sequence Read Archive database under accession number PRJNA1104563.
